# Three Distinct Circovirids Identified in a Tapeworm Recovered from a Bobcat (*Lynx rufus*)

**DOI:** 10.3390/v17060745

**Published:** 2025-05-23

**Authors:** Ayla Žuštra, April Howard, Katie Schwartz, Ron Day, Jaclyn Dietrich, Caroline Sobotyk, Simona Kraberger, Arvind Varsani

**Affiliations:** 1 Biodesign Center for Fundamental and Applied Microbiomics, School of Life Sciences, Center for Evolution and Medicine, Arizona State University, Tempe, AZ 85287, USA; azustra@asu.edu; 2 Arizona Game and Fish Department, 5000 W. Carefree Highway, Phoenix, AZ 85086, USA; ahoward@azgfd.gov (A.H.);; 3 48351 State Route 255, Sardis, Monroe, OH 43946, USA; 4 Department of Pathobiology, School of Veterinary Medicine, University of Pennsylvania, 3900 Delancey St., Philadelphia, PA 19104, USA; jaclyndi@vet.upenn.edu (J.D.);; 5 Structural Biology Research Unit, Department of Integrative Biomedical Sciences, University of Cape Town, Cape Town 7925, South Africa

**Keywords:** bobcat, *Lynx rufus*, *Circoviridae*, *Circovirus*, *Cyclovirus*, helminth, *Taenia*, *Physaloptera*, *Metathelazia*

## Abstract

Bobcats (*Lynx rufus*) are an iconic North American predator; however, there is limited knowledge regarding their associated parasites. In this case study, we used a metagenomic approach to identify associated viruses and helminth species from a deceased bobcat. We determined the full mitochondrial genome of the bobcat and three helminths, i.e., tapeworm (*Taenia* sp.), stomach worm (*Physaloptera* sp.), and lung worm (*Metathelazia* sp.). Furthermore, we identified four circovirids; two (identified in a tapeworm and fecal swab) are members of the genus *Circovirus* and share 96.7% genome-wide identity between isolates and 87.4–88.6% identity with members of the species *Circovirus miztontli*. These appear to infect vertebrate species common to the Sonoran Desert, which could be a rodent preyed upon by the bobcat, and/or bobcat itself. The other two circovirids are novel members of the genus *Cyclovirus* (both identified in a tapeworm), one sharing 99.8% with those in the species *Cyclovirus misi* from a rodent and the other <67.3% with all other *Cycloviruses*. Our data support that these two *Cycloviruses* are likely tapeworm-infecting; however, more studies are needed to confirm the host. These findings enhance our understanding of viruses and helminths in bobcats, emphasizing the need for further research to unravel the ecology of parasites in these elusive predators.

## 1. Introduction

Bobcats (*Lynx rufus*) are widespread in North America, with their distribution spanning from southern Canada, most of the contiguous United States of America (USA), to Mexico [1]. Within the USA, the population size estimates in 2010 indicated 2.3 to 3.5 million [2], with their natural habitat range including forest, grassland, coastal swamp, agricultural zone, and desert ecosystems [3,4]. Despite increased urbanization in the aforementioned areas, bobcats have, in many cases, adapted to living in these urban areas, resulting in more frequent interactions with humans, domestic animals, and other urbanized wildlife [5,6,7,8,9]. Bobcats are found across Arizona, USA, both in urbanized and rural areas, including the Sonoran Desert [10]. Despite bobcats being considered solitary as adults, interactions between young kittens and their mothers, environmental factors, trophic cascades, and conflict or social interactions serve as opportunities for the acquisition of pathogens such as helminths and viruses [11,12].

Bobcats are known to harbor epizootic and zoonotic endoparasites, including helminths in the phylum Nematoda (nematodes) and Platyhelminthes (cestodes and trematodes) [13]. Of the cestodes (commonly referred to as tapeworms), species from the genera *Spirometra*, *Mesocestoides*, and *Taenia* are the most commonly identified ones in bobcats [14,15]. Adult tapeworms are found in the intestinal tract of their definitive host, while larval stages can infect other parts of the body, particularly soft tissues and skeletal muscles of intermediate hosts [16]. Of the nematodes, those in the genera *Physaloptera* (commonly referred to as stomach worms), *Metathelazia* and *Vogeloides* (commonly referred to as lungworms), *Cyathospirura* (unspecified common term), *Toxascaris* and *Toxocara* (commonly referred to as roundworms), and *Spirocerca* (esophageal worms) have been found in bobcats [13,17,18]. *Physaloptera* spp. are found in the gastrointestinal tracts of a variety of hosts, ranging from amphibians, birds, reptiles, to mammals, and infect a variety of intermediate arthropod hosts [19]. *Metathelazia* spp. are lungworms that infect the lung parenchyma, bronchi, and bronchioles in carnivores, though their life cycle remains largely unknown [20]. Wild felids most likely acquire these parasites either through the environment or via their prey, infected with these helminths [14].

Several viruses have been identified in bobcats, including viruses belonging to the families *Anelloviridae* [21], *Circoviridae* [22,23], *Herpesviridae* [24,25], *Papillomaviridae* [26], *Paramyoxiviridae* [27], *Parvoviridae* [28], *Polyomaviridae* [29], *Retroviridae* [30,31,32], *Rhabdoviridae* [33], and *Smacoviridae* [34]. Some of these viruses are not associated with disease outcomes.

Viruses in the family *Circoviridae* (*Cressdnaviricota* phylum) have circular single-stranded DNA genomes which encode a capsid protein (CP) and a replication-association protein (Rep). Within the *Circoviridae* family, there are two genera, *Circovirus* and *Cyclovirus*, which differ primarily in the orientation of the Rep and CP protein-coding genes relative to the origin of replication [35,36,37]. Additionally, the Rep and CP proteins of *Circoviruses* and *Cycloviruses* are phylogenetically distinct [37]. Some members of the *Circovirus* genus are known to cause severe disease, such as beak and feather disease, in primarily psittacine species [38,39], and postweaning multisystemic wasting syndrome in porcine species [40,41]. Both *Circoviruses* and *Cycloviruses* have previously been identified from wild bobcat scat, *Circoviruses* from those collected in the Sonoran Desert, Mexico, and California, USA, and a *Cyclovirus* from the latter [23,42].

Here, we undertake a case study on a deceased wild bobcat, expanding on what is known about the helminths and viruses associated with bobcats.

## 2. Materials and Methods

### 2.1. Sample Collection

A single deceased male bobcat (whose cause of death was deemed to be the result of a broken rib that punctured its diaphragm) was found northeast of the greater Phoenix area (33.77250 N, 111.49405 W), Arizona (USA) on 31 January 2024. A necropsy was performed the same day, and the following samples were collected: a rectal swab, spleen, and liver, and helminths: from the stomach one nematode specimen (stomach worm) and one cestode specimen (tapeworm), and the lungs one nematode specimen (lungworm). The rectal swab was stored in UTM buffer at 4 °C, the organ samples at −20 °C, and the helminths were stored in 70% ethanol until processing. The ethanol was evaporated off prior to extraction.

### 2.2. Nucleic Acid Extractions and Metagenomics

Small segments from the middle section of the tapeworm, liver, and spleen, as well as one whole lungworm and one stomach worm, were separately homogenized in 400 μL of SM buffer. These were centrifuged at 10,000 rpm for 10 min and 200 μL of homogenate was aliquoted into a new 1.7 mL tube. A total of 200 μL of the UTM buffer from the rectal swab was also aliquoted to a new 1.7 mL tube. These were then utilized for viral DNA extraction using the High Pure Viral Nucleic Acid (Roche Life Sciences, Basel, Switzerland). An amount of 2 μL of the viral DNA extraction was used to enrich circular DNA molecules using rolling-circle amplification (RCA) with the phi29 kit (Watchmaker, Stoneham, MA, USA). In total, 200 μL of the homogenate from each sample was also utilized for total RNA extraction using the QIAwave RNA Plus mini kit (Qiagen, Germantown, MD, USA).

For each DNA sample, 15 μL of RCA product was combined with 15 μL of viral DNA extraction product. High-throughput sequence libraries for these samples were prepared using the Illumina DNA LP (M) Tag 96 sample preparation kit (Illumina, San Diego, CA, USA) these were sequenced at Psomagen Inc. (Rockville, MD, USA) on an Illumina NovaseqX Psomagen Inc. (USA). A total of 11 μL of viral RNA was used to generate libraries with the Zymo-Seq RiboFree Total RNA Library Kit (Irvine, CA, USA) and sequenced at the Zymo Research sequencing core on an Illumina Sequencer. The Illumina sequence raw reads were trimmed using Trimmomatic v.0.39 [43], and then, the reads were *de novo* assembled using MEGAHIT v.1.2.9 [44]. Contigs were analyzed for virus-like sequences using BLASTx against a viral RefSeq database (release 220). Contigs > 1000 were analyzed with CenoteTaker2 [45] to identify and annotate viral-like genomes; additionally, complete circular genomes based on terminal redundancy were also identified. Circular contigs sharing similarities with members of the *Circoviridae* family were identified, and the open reading frames (ORFs) were identified; these ORFs were manually checked. We also screened the contigs for host mitochondrial genomes using Diamond BLASTx [46] with a mitochondrial RefSeq database (release 220). The mitochondrial genomes of the bobcat and three helminths (one cestode and two nematodes) were annotated using the MITOS server [47], and manually checked against the most closely related mitochondrial reference genomes.

### 2.3. Mitochondrial Sequence Analyses

We constructed a dataset of mitochondrial sequences from the bobcat from this study together with all other bobcat mitochondrial genome sequences available in GenBank (GQ979707, KP202285, CM039064, KR132584), representatives from other members of the *Lynx* genus: the Canada lynx (*Lynx canadensis*), Eurasian lynx (*Lynx lynx*), and Iberian lynx (*Lynx pardinus*) and one from a mountain lion (*Puma concolor*) as an outgroup. For the analysis of the cestode mitochondrial sequences, a dataset from the Taeniidae family (*Taenia* genus), the cestode from this study, and that of *Hydatigera taeniaeformis* as an outgroup was constructed. Finally, for the analyses of the two nematodes’ mitochondrial sequence part of the *Spiruromorpha* infraorder, we constructed a dataset with representatives from the *Spiruromorpha* infraorder and *Wellcomia siamensis* as an outgroup. From the cestode and nematode datasets, we extracted the *coxI* gene, subunit I of mitochondrial cytochrome c oxidase, sequences for additional analyses.

Sequences in these datasets were individually aligned using MAFFT [48] and the alignments were used to infer maximum-likelihood phylogenetic trees using PhyML 3 [49], with the best substitution models determined using jModelTest2 [50]. The nucleotide substitution model determined for the bobcat mitochondrial dataset was the HKY+G model, for the nematode and cestode datasets was GTR+G+I, for the cestode *coxI* dataset was TN93+G+I, and for the nematode *coxI* dataset was GTR+G.

Branches with <0.8 aLRT support in the mitochondrial sequence phylogenetic trees were collapsed in TreeGraph 2 [51], and the phylogenies were visualized and edited in iTOL [52].

### 2.4. Circovirus Sequence Analyses

A dataset of *Circovirus* sequences of all representative classified species [35], together with sequences from this study, was constructed. The genome sequences were aligned using MAFFT [48] and maximum-likelihood phylogenetic trees inferred using PhyML 3 [49], with the GTR+G+I nucleotide substitution model, determined using jModelTest [53], for the *Circoviruses* and GTR+G+I for *Cycloviruses*. In addition to this, from genome datasets, we extracted the Rep and CP ORFs. These ORF datasets were translated and aligned using MAFFT [48]. The amino acid sequence alignment was trimmed using TrimAL [54] and this was then used to infer maximum-likelihood phylogenetic trees using PhyML 3 [49], with the RtREV+G+I substitution model determined as the best fit model using ProtTest 3 [55]. Branches with <0.8 aLRT support in the Rep and CP phylogenetic trees were collapsed in TreeGraph 2 [51], and the phylogenies were visualized and edited in iTOL [52]. The full genome nucleotide sequence, and Rep and CP amino acid sequence pairwise identities for the *Circoviruses* were determined using SDT 1.2 [56]. Branches with <0.8 aLRT support were collapsed in TreeGraph 2 [51], and the phylogenies were visualized and edited in iTOL [52].

*Circoviruses* identified in the study, together with the most closely related viral sequences available in GenBank, were further analyzed using Clinker [57].

## 3. Results and Discussion

A necropsy was performed on a deceased male bobcat that was found to be severely emaciated. The cause of death was later determined to be a result of a punctured diaphragm caused by a broken rib from an unknown cause. As part of the diagnostic examination, samples were sent to determine the presence of a suite of known feline-infecting pathogens, canine distemper virus, plague, and tularemia, none of which were detected.

To complement the tests for feline-infecting pathogens, a metagenomic approach was used to identify known and novel viruses as well as host/source mitochondrial genomes from a rectal swab, liver and spleen samples, and in tapeworms, stomach worms (sampled from the stomach), and lungworms (sampled from the lung). From these samples, we determined the genomes of four circovirids (family *Circoviridae*), two of which are members of the genus *Circovirus* and two of *Cyclovirus*, and the mitochondrial genomes of the bobcat and the three helminths.

### 3.1. Bobcat Mitochondrial Genome

**Figure 1:**
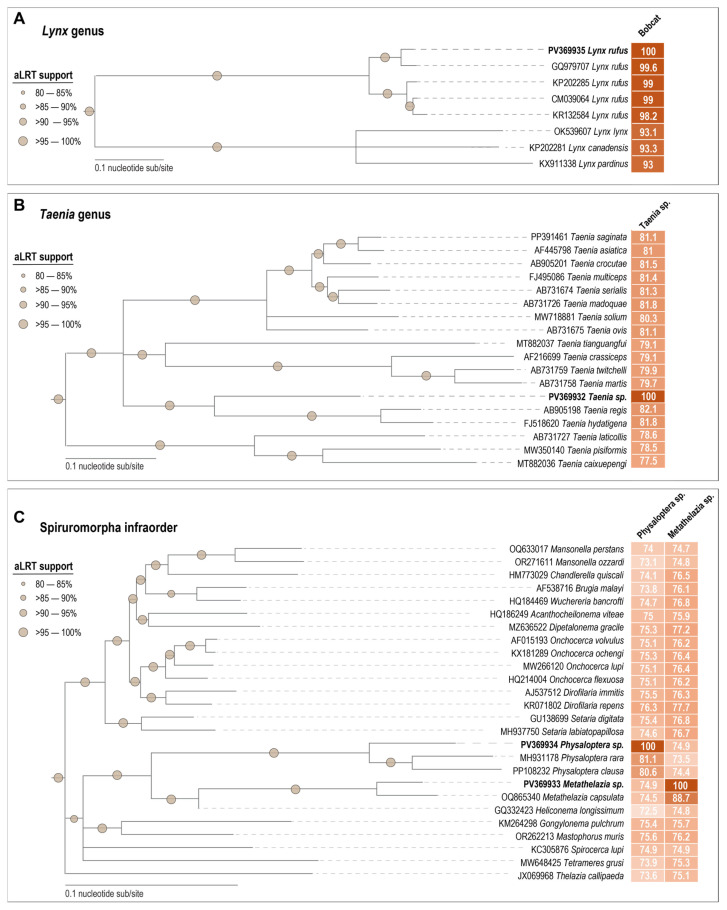
Maximum-likelihood phylogenetic trees of (**A**). Bobcat (*Lynx rufus*) mitochondrial genome sequences available in GenBank, together with those from this study (bold font) and species representatives from the genus *Lynx*. (**B**). Cestode (tapeworm) full mitochondrial genome from this study (bold font) and species representative from the genus *Taenia*. (**C**). Nematode full mitochondrial genomes from this study (bold font) and species representatives from the *Spiruromorpha* infraorder.

The bobcat mitochondrial genome is 17,114 bp (PV369935) and phylogenetically clusters in a clade with a mitochondrial genome (GQ979707) identified from a bobcat from Texas, USA, sharing 99.6% genome-wide pairwise identity (Figure 1A). These two genomes are sister clades with the other three available mitochondrial genomes: from two bobcats (KP202285) [58] and KR132584 [59] from an unspecified location in North America, and a genome isolated from a bobcat (CM039064) [60] from California, USA. These genomes share 98.2–99.8% genome-wide pairwise identity (Figure 1A and Supplementary Data S1). These share 93–93.3% genome-wide pairwise identity with representative mitochondrial genomes from other species in the genus *Lynx* (Figure 1A). Very little bobcat mitochondrial genetic information is currently available, and therefore, mitochondrial data from the deceased bobcat from Arizona help towards getting a better handle on the genetic diversity of bobcats within the context of their maternal lineages across North America.

### 3.2. Helminth Mitochondrial Genomes

The complete mitochondrial genome from the tapeworm is 13,534 nts (PV369932) in length and falls in the family Taeniidae, genus *Taenia* [61]. Phylogenetic analyses of the mitochondria of members of the genus *Taenia* show that the tapeworm from this study sits basal to a clade of mitochondrial genome sequences from *Taenia regis* (AB905198) recovered from an African Lion from Kenya [62] and *Taenia hydatigena* (FJ518620) recovered from a domestic dog in China [63] (Figure 1B). The life cycle of these *Taenia* species includes intermediate hosts, often omnivores or herbivores (rodents or lagomorphs) that ingest the *Taenia* eggs, which then develop into larvae in their organs. The intermediate hosts are then preyed upon by the definitive host, often a carnivore (including felids and canids), where the adult tapeworm inhabits the small intestine, continuing its lifecycle and releasing eggs via the gut into the environment [64]. *Taenia regis* to date has been shown to be endemic to felids in Africa [62], while *T. hydatigena* is commonly found in ruminants, pigs, canids, and felids worldwide [63,65,66,67,68]. The mitochondrial genome pairwise analyses show the bobcat-derived *Taenia* shares 82.1% and 81.8% similarity with that of *T. regis* and *T. hydatigena*, respectively (Figure 1B). For some *Taenia* species, only the *coxI* hypervariable region is available in the public database; therefore, additional analyses of this region show that the one from the bobcat-derived *Taenia* is most closely related to *Taenia rileyi* (Supplementary Figure S1), sharing 96.5% pairwise identity. Further, this is the most common tapeworm species found in bobcats across North America [14], therefore supporting *T. rileyi* as the most likely species for this helminth. However, more information about morphology, life cycle, and host range would help support a more concrete identification.

The mitochondrial genome of the stomach worm recovered from the stomach of the bobcat is 13,723 nts in size (PV369934). Phylogenetically, the stomach worm mitochondrial genome sequence clusters with that of two members of the genus *Physaloptera* (family Physalopteridae), i.e., *Physaloptera clausa* (PP108232) from a European hedgehog (*Erinaceus europaeus*) from China, and *Physaloptera rara* (MH931178) from a canid in the USA, sharing 80.6% and 81.1% mitochondrial genome-wide pairwise identity, respectively (Figure 1C and Supplementary Data S1), showing this bobcat roundworm falls in the genus *Physaloptera*. *Physaloptera clausa* is a nematode that has almost exclusively been identified thus far to parasitize European hedgehogs in Italy, Germany, Turkey, Poland, and in the Middle East [69,70,71,72,73], and is transmitted via an arthropod intermediary host [74]. Severe *P. clausa* parasitic infection in animals can lead to weight loss, anemia, and cachexia. It is also a potential vector for leptospirosis, which is of spillover risk to other animals, including humans [69]. Generally, for roundworms, there is typically only a singular host, and the roundworm eggs are excreted, develop into larvae, and are ingested by the host, where they burrow into the gut lining [75]. *P. rara* has been found in a variety of carnivores, including dogs, coyotes, raccoons, foxes, wolves, and felids [76] in the USA, Canada, and Japan [77,78,79]. Much like *P. clausa*, and other *Physaloptera* sp., *P. rara* is often transmitted via an arthropod intermediary and parasitizes the stomach of its host, where burrowing/movement can cause lesions throughout the gastrointestinal tract [80]. *Physaloptera praeputialis* is a common stomach worm in felids [81,82]; however, no mitochondria or *coxI* gene sequences are available in the public database, thus we are not able to analyze this with our data. Given the similarity between the bobcat-derived stomach worm sequence from this study and the two *Physaloptera* sp. mitochondrial sequences available in public databases (*P. rara* and *P. clausa*), of 81.1% and 80.6% genome-wide identity, respectively (Figure 1C), which is below the threshold shown for designation between species in other genera within the family Physalopteridae (Supplementary Data S1), we are thus only able to identify the stomach worm to a genus level *Physaloptera* sp. without further morphology, life cycle and host range information no clear species can be assigned.

The third helminth mitochondrial genome we determined is 13,609 nts in length (PV369933) of a lungworm which was recovered from the lung of the bobcat. Based on the phylogenetic analyses, the mitochondrial sequence of this lungworm is most closely related to that of *Metathelazia capsulata* (family Pneumospiruridae) (OQ865340) [83] (Figure 1C and Supplementary Data S1), sharing 88.7% mitochondrial genome-wide identity. *Metathelazia capsulata* parasitizes the bronchi and bronchioles of wild carnivores, namely foxes, badgers, and polecats; however, the definitive life cycle is still not fully known [20,84,85]. Lungworms are ingested in the larval stage, either by an intermediate host such as a mollusk or rodent [86], which is then predated on by the final host, and lives in the lungs or pulmonary arteries, though the specific life cycle of *M. capsulata* is unknown [20]. Members of the genus *Metathelazia* have been described worldwide [87,88]. Altogether, it indicates that this lungworm we found in the bobcat most likely belongs to the species *M. capsulata*, given the genetic similarity and the hosts it typically infects. However, given the limited sequence data on other members of the *Metathelazia* genus, a definitive species cannot be assigned.

### 3.3. Circovirid Genomes

We identified four circovirid (family *Circoviridae*) genomes, three in the bobcat-derived tapeworm (*Taenia* sp.) and one from the bobcat fecal sample. In the four genomes, we identified the conserved nonanucleotide sequence TAGTATTAC (PV339922, PV339923) for the two members of the genus *Circovirus*, and CAGTGTTAC (PV339925) and AAGTATTAC (PV339924) for the two members of the genus *Cyclovirus*. These nonanucleotide sequences are part of the stem loop for the initiation of replication of the viruses. The Reps of the two *Cycloviruses*, *Calfel cyclovirus* (PV339925) and *Tenaz cyclovirus* (PV339924), from the *Taenia* sp. have introns of 31 and 71 nts, respectively. In the Rep of the circovirids, we identified the conserved rolling circle replication endonuclease motifs (motif I, II, and II) and the superfamily 3 helicase motifs (Walker A and B, motif C) [35,89], which are summarized in Table 1

### 3.4. Circovirus

The two circovirids: PV339922, genome size 2162 nts from the tapeworm; and PV339923, genome size 2156 nts from the fecal sample, share 96.7% genome-wide pairwise identity and are referred to here as lyrufec (*Lynx rufus* fecal associated) *Circovirus*, and are members of the genus *Circovirus*. These two genomes share 87.4–88.6% genome-wide pairwise identity (Supplementary Data S2) with the *Chaetfec circovirus* (OM154926) from a rodent (*Chaetodipus* sp.) fecal sample collected in Arizona, USA [90], and *Sonfela circovirus* 2 (MT610106) from a bobcat fecal sample collected in the Sonoran Desert, Mexico [42] and classified in the species *Circovirus miztontli*. Based on the species demarcation criteria (80% genome-wide pairwise identity threshold) for members of the family *Circoviridae* [35,37], the two members of the Circovirus genus share > 87.6% pairwise identity with other members of the species *Circovirus miztontli* and thus belong to this species. It is important to highlight that four members of the species *Circovirus miztontli* have been identified from animal samples, all collected in the Sonoran Desert in the southwest of North America, and therefore are infecting an organism that is common to this region.

**Figure 2:**
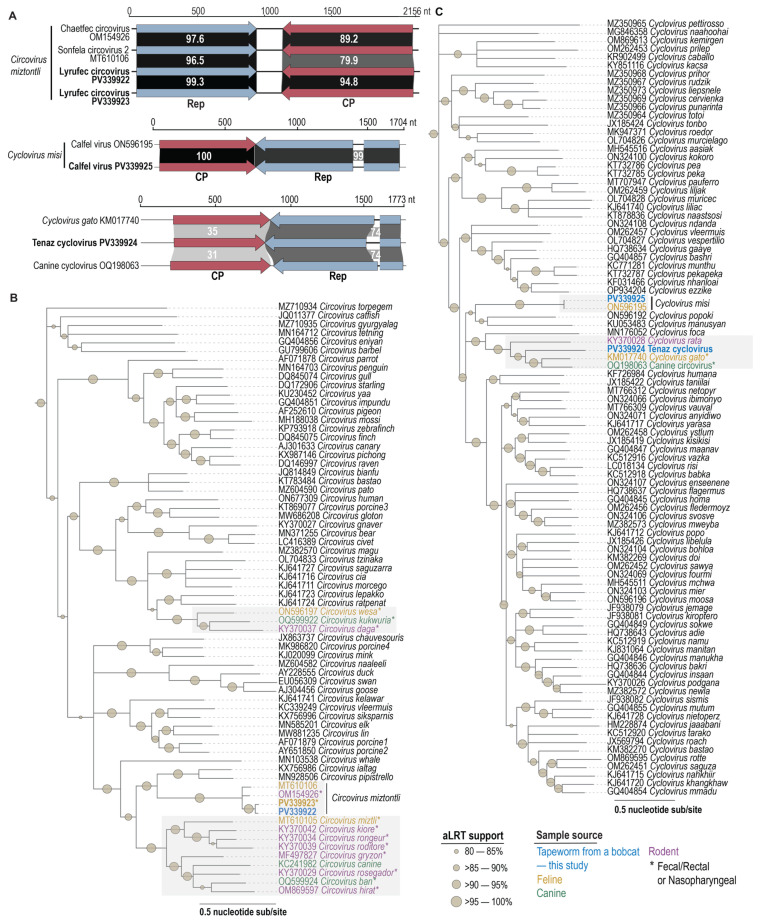
(**A**). Linearized genome illustration of identified *Circoviruses* and *Cycloviruses* in the study, with Rep and CP amino acid pairwise identities to the nearest neighbor shown. (**B**). Maximum-likelihood phylogenetic tree of the genome sequences of representative members of the genus *Circovirus*. (**C**). Maximum-likelihood phylogenetic tree of the genome sequences of representative members of the genus *Cyclovirus* and those identified in this study. The grey boxes highlight clades with sequences from this study, with bolded names denoting those from this study. Color key indicates sample type within highlighted clades. “*” is shown in the sample source information on the figure.

**Figure 3:**
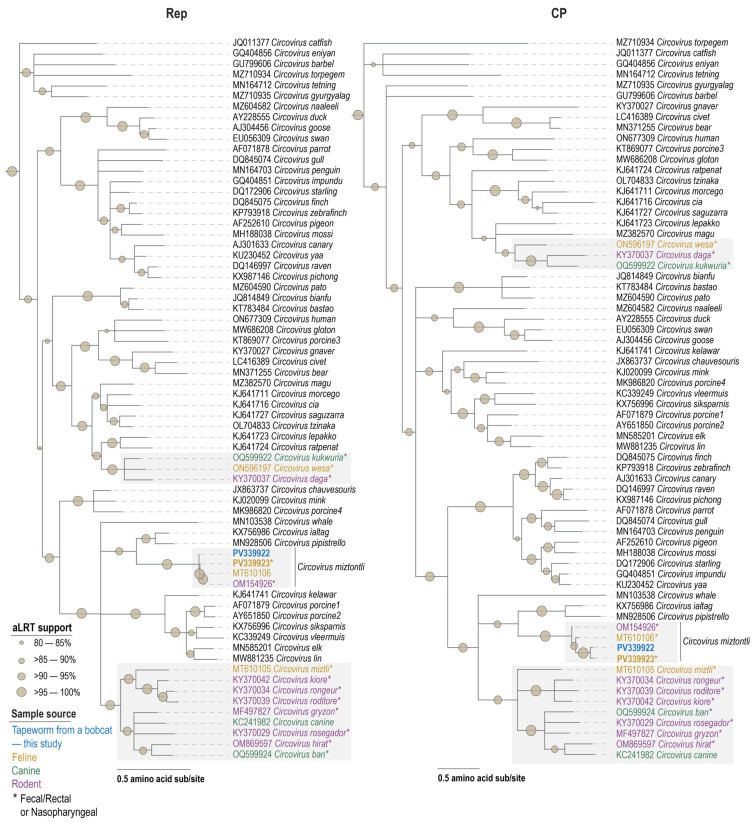
Maximum-likelihood phylogenetic tree of Rep and CP amino acid sequences of representative members of the genus *Circovirus*. The phylogenetic trees are rooted with representatives from the genus *Cyclovirus*. The grey boxes highlight clades with sequences from this study, with bolded names denoting those from this study. Color key indicates sample type within highlighted clades. “*” is shown in the sample source information on the figure.

Given the high sequence identity at a genome level, it is not surprising that the Reps and CPs of the two new members of the species *Circovirus miztontli* from this study share 99.3% and 94.8% amino acid identity between them, respectively (Figure 2). When compared to those of the other two members of this species, they share 95.8–97.0% Rep amino acid and 79.9–80.5% CP amino acid identities (Supplementary Data S2). In the phylogenetic analyses of the genomes, Reps and CPs all cluster together (Figure 2 and Figure 3). In general, the Rep of the four members of the species *Circovirus miztontli* share 41.8–64.1% identity with that of other representatives of the genus *Circovirus* and 22.1–47.3% identity for the CP. Relative to the Reps, the CPs are less conserved; however, it is interesting to note that a significant number of *Circoviruses* that have been identified from feline, canine, and rodent samples cluster in a polyphyletic clade (Figure 2). The felid-, canid-, and rodent-derived circoviral genomes in this polyphyletic clade are all from fecal, rectal, or nasopharyngeal swab samples [90,91,92,93,94,95], with the exception of the members of the species *Circovirus canine* that have been identified in tissue [96,97,98,99,100].

### 3.5. Cyclovirus

The other two circovirids: *Calfel cyclovirus* (PV339925; 1704 nts) and *Tenaz cyclovirus* (PV339924; 1771nts), were both identified in the bobcat-derived tapeworm (*Taenia* sp.), and share 59.8% genome-wide identity between themselves (Supplementary Data S3). These two viruses are members of the genus *Cyclovirus*. PV339925 is most closely related to the genome *Calfel virus* LSF31_cyc420 (ON596195) from a bobcat scat sample collected in California, USA [23], sharing 99.8% genome-wide pairwise identity. Since *Calfel virus* LSF31_cyc420 is classified in the species *Cyclovirus misi*, PV339925 is also a member of this species (Supplementary Data S2). On the other hand, PV339924 (*Tenaz cyclovirus*) represents a member of a new species based on the species demarcation criteria for the family *Circoviridae* [35,37].

**Figure 4:**
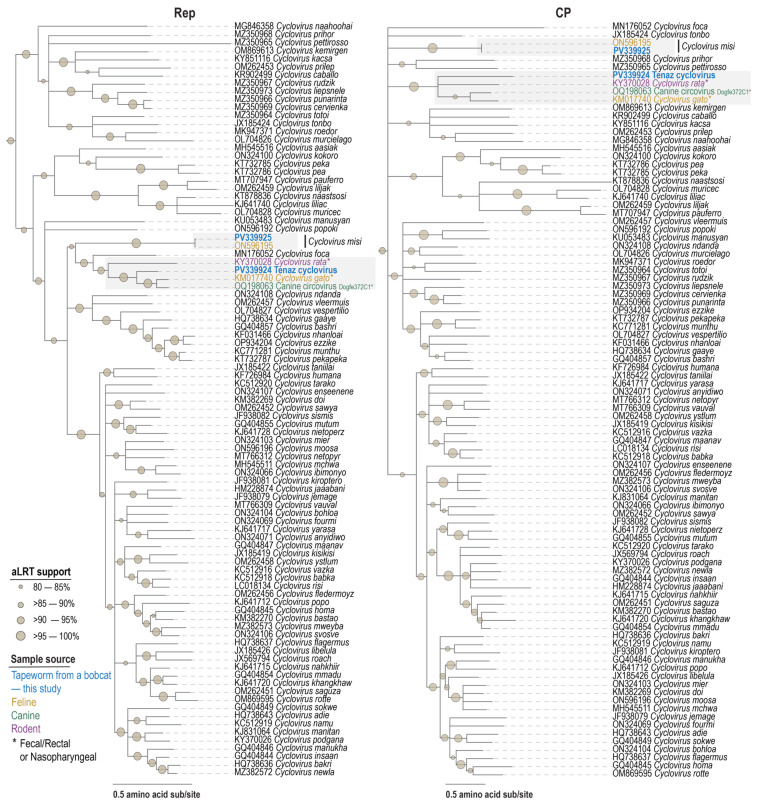
Maximum likelihood phylogenetic tree of Rep and CP sequences of representative members of the genus *Cyclovirus*. The phylogenetic tree was rooted with representative sequences from the genus *Circovirus*. The grey boxes highlight clades with sequences from this study, with bolded names denoting those from this study. Color key indicates sample type within highlighted clades. “*” is shown in the sample source information on the figure.

Analyses of the Rep and CP amino acid sequences of *Calfel cyclovirus* (PV339925) show that they share 99.3% and 100% identity with those of *Calfel virus* LSF31_cyc420 (ON596195), respectively (Supplementary Data S3). This close relationship is also supported in the phylogenetic trees of the genomes, Rep and CP (Figure 2 and Figure 4). When compared to other viruses, their Reps and CPs share, these two members of the species *Cyclovirus misi* share 33.9–52.4% Rep amino acid and 14.1–32.5% CP amino acid identity (Figure 4 and Supplementary Data S3).

The Reps and CPs of *Tenaz cyclovirus* (PV339924) are phylogenetically most closely related to those of *Feline cyclovirus* (KM017740; species *Cyclovirus gato*) from a domestic cat [101], *Canine circovirus* Dogfe372C (OQ198063; unclassified *Cyclovirus*) from a domestic dog (ref), and rodent-associated *Cyclovirus* 1 RtRf-CV-2/YN2013 (KY370028; species *Cyclovirus rata*) from buff-breasted rat (*Rattus flavipectus*) [92], sharing 57.6–74.6% Rep amino acid and 29.9–40.0% CP amino acid identity (Figure 4 and Supplementary Data S3).

### 3.6. Circovirid Host

We did not identify any reads from DNA data from the bobcat organ samples that mapped the two *Circoviruses* that are members of the species *Circovirus miztontli* identified in the *Taenia* sp. and the feces samples. Furthermore, we did not find any reads from our RNA sequencing data that mapped to these genomes, suggesting these viruses were not being transcribed in the tissue samples we collected. Thus, the more parsimonious hypothesis is that these two *Circoviruses*, including the *Taenia* sp., are likely prey derived from a rodent that was predated on shortly before the bobcat’s death.

**Figure 5:**
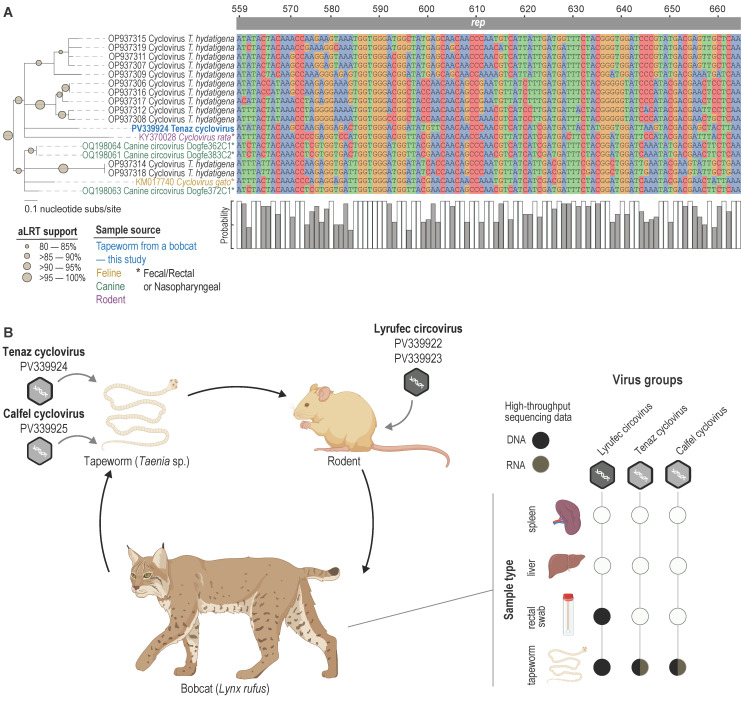
(**A**). Maximum-likelihood phylogenetic tree of the region of the Rep-coding sequence of *Tenaz cyclovirus* (PV339924) and other related sequences of *Cycloviruses*. An alignment spanning 104 nt is shown with the relative nucleotide probability. (**B**). Virus–host interaction hypotheses of the three lineages of *Circoviruses*: *Lyrufec circoviruses* (species *Circovirus miztontli*) and the two *Cycloviruses*, *Tenaz cyclovirus* (PV339924) and two *Calfel cyclovirus* (PV339925), species *Cyclovirus misi*. Created in https://BioRender.com. “*” is shown in the sample source information on the figure.

On the other hand, we identified reads from the RNA sequencing data from *Taenia* sp. that map to both the *Cyclovirus* genomes, suggesting transcription, which would indicate the virus is replicating in the Tapeworm; however, further studies using approaches such as in situ hybridization are needed to confirm this. A BLASTn [102] analysis of the *Cycloviruses* from this study showed that sequences of *Tenaz cyclovirus* (PV339924) share nucleotide sequence similarities to *Cyclovirus* sequences from fecal samples of a domestic cat, dogs, and a rodent, as well as *Cyclovirus* sequences in the Rep coding region identified in *Taenia hydatigena* from dogs in China [92,101,103] (Figure 5A). Additionally, the probabilities of the nucleotides at specific positions in the alignments highlight the similarities between the *rep* sequences. Zhang et al. [103] hypothesize that these *Cycloviruses* infect the canine-derived *T. hydatigena* in their study. This, together with our findings, supports that these lineages of *Cycloviruses* may infect *Taenia* species that parasitize rodents, canids, and felids. Patterson et al. [104] identified *Cyclovirus* genomes in fecal samples of Weddell seals, and these have similarities to Rep-like sequences in the genome assemblies of a tapeworm Spirometra tapeworm, hypothesizing that these *Cycloviruses* likely infect tapeworm species parasitizing the Weddell seals. Further, endogenized Rep-like sequences have been found in various animals [105,106], and these may help identify putative host lineages of viruses identified via metagenomic approaches from environmental samples as well as tissue samples that have mixed infection with various parasites.

Thus, given the presence of the two *Circoviruses* (species *Circovirus miztontli*) (PV339922 and PV339923) in the tapeworm and rectal swab, and the presence of the two *Cycloviruses*, *Tenaz cyclovirus* (PV339924) and *Calfel virus* (PV339925) solely in the tapeworm, we propose the following putative movement/infection hypotheses (Figure 5B). We propose that the *Circoviruses*, identified as the species *Circovirus miztontli*, are infecting the rodent prey, and are found in the tapeworm because the tapeworm present in the bobcat originated from the prey upon infected rodent, and is later excreted by the bobcat hence this was also identified in the fecal swab. Both *Cycloviruses* were found exclusively in the tapeworm and in both the RNA and DNA sequencing, implying active replication of the virus in the tapeworm, and thus appears to be replicating in the tapeworm directly rather than the bobcat host; however, further studies are needed to confirm the host of both *Cycloviruses*.

## 4. Conclusions

In this study, via metagenomics, we determined the mitochondrial genomes of three helminths: tapeworm, stomach worm, and a lungworm, recovered from a deceased bobcat, and four virus genomes that are members of the family *Circoviridae*. We were able to successfully determine the bobcat mitochondrial genome, as well as that of the three parasites determined to be *Taenia* sp., *Physaloptera* sp., and *Metathelazia* sp. Given the species designation for the *Taenia* and *Physaloptera* genera, and potentially *Metathelazia*, it is likely the cestodes and nematodes in the study could represent novel insights into parasite biology; further morphological and life cycle analyses would be necessary to confirm this. Despite this challenge, these findings broaden the genetic data available for these organisms. We also identified three viruses in the *Taenia* sp. tapeworm from the bobcat, one of which represents a new species. *Lyrufec circovirus* (PV339922 and PV339923) was only detected in the tapeworm and bobcat rectal swab, neither organ sample, nor was it present in the RNA sequencing data, suggesting the virus was not replicating in the bobcat or the tapeworm. Whereas the *Cycloviruses* were only detected in the tapeworm (Figure 5B), in both DNA and RNA sequence data.

Taken together with phylogenetic relationships (Figure 2, Figure 3 and Figure 4), similarities to those of other feline, canine, and rodent Rep proteins, and *rep* nucleotide sequences from *Taenia hydatigena* and other vertebrate fecal samples supports our hypothesis that the *Circoviruses* are likely prey-derived or/and bobcat infecting, whereas the two *Cycloviruses* are infecting the tapeworm (Figure 5B). If in fact, these are tapeworm-infecting *Cycloviruses*, it broadens how we can investigate tapeworm life cycle and trophic interactions [107] using viruses as a proxy. Previous studies have shown *Entamoeba* and *Giardia* infecting cressdnaviruses in human samples clearly suggesting a proxy for using these viruses to identify their host’s active infection of mammals [108,109].

This study highlights the complex interactions between hosts, prey, parasitic helminths, and their associated *Circoviruses*, shedding light on the underexplored virosphere of parasitic organisms. Continued research in this area is needed to provide a better understanding of how viruses influence helminth biology, life cycles, and predator/prey host–helminth dynamics.

## Figures and Tables

**Table 1:** Summary of the rolling-circle replication (RCR) endonuclease motifs (I, II, III), the superfamily 3 helicase motifs (Walker A and B, motif C), and the Arg finger, found in replication-associated proteins (Rep) of the four circovirids identified in this study.

**Species**	**Virus**	**Accession**	**Motif I**	**Motif II**	**Motif III**	**Walker A**	**Walker B**	**Motif C**	**Arg Finger**
*Circovirus miztontli*	*Lyrufec circovirus*	PV339922	AFTLNN	PHLQG	DNKKYCSK	GPPGTGKSRECL	IMDDF	ITSN	ALFRRI
*Circovirus miztontli*	*Lyrufec circovirus*	PV339923	AFTLNN	PHLQG	DNKKYCSK	GPPGTGKSRECL	IMDDF	ITSN	ALFRRI
*Cyclovirus misi*	*Calfel cyclovirus*	PV339925	VFTHFN	KHLQG	DNQKYCSK	GEPGTGKSKTAL	IIDDF	ITSN	AIKRRC
unclassified	*Tenaz cyclovirus*	PV339924	CFTLNN	PHLQG	QNRTYCSK	GPPGVGKSRRAY	IIDDY	ITSN	AIERRC

## Data Availability

Sequence data have been deposited to the NCBI Sequence Read Archive under accession number BioProject: PRJNA1236407; BioSample: SAMN47387171, SAMN47387172, SAMN47387173, SAMN47387174, SAMN47387175, SAMN47387176; SRA: SRR32702192, SRR32702193, SRR32702194, SRR32702195, SRR32702196, SRR32702197. The *Circovirus* and *Cyclovirus* genomes have been deposited in GenBank under accession #s PV339922-PV339925 and PC369932-PV369935.

## Supplementary Materials

The following supporting information can be downloaded at https://www.mdpi.com/article/10.3390/v17060745/s1, Supplementary Figure S1: Maximum-likelihood phylogenetic tree (A) cestode (tapeworm) *coxI* hypervariable region from this study (bold) and species representative from the genus *Taenia*. (B) Nematode *coxI* hypervariable region from this study (bold) and species representatives from the *Spiruromorpha* infraorder; Supplementary Data S1: Percentage pairwise identity matrices of the mitochondrial sequences identified in this study with those of closely related sequences. Bold text denotes those from this study; Supplementary Data S2: Percentage pairwise identity matrices of the full genome, as well as the coding region (nucleotide and protein sequences) of the sequences in the genus *Circovirus*, including the two from this study. Bold text denotes those from this study; Supplementary Data S3: Percentage pairwise identity matrices of the full genome, as well as the coding region (nucleotide and protein sequences) of the sequences in the genus *Cyclovirus*, including the two from this study. Bold text denotes those from this study.
