# Supplementary figures and images for: Three Distinct Circovirids Identified in a Tapeworm Recovered from a Bobcat (Lynx rufus)
Source: Viruses. 2025 May 23;17(6):745. doi: 10.3390/v17060745 (PMC12197738; doi:10.3390/v17060745)

A

Taenia genus

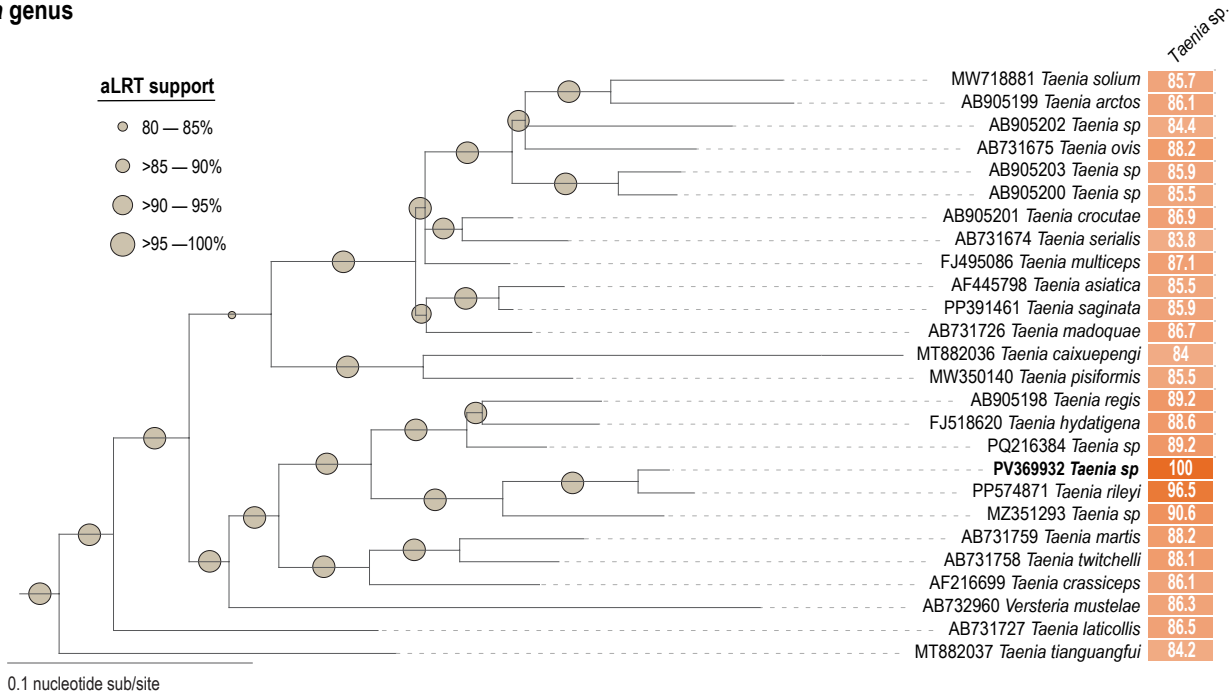

B

Spiruromorpha infraorder

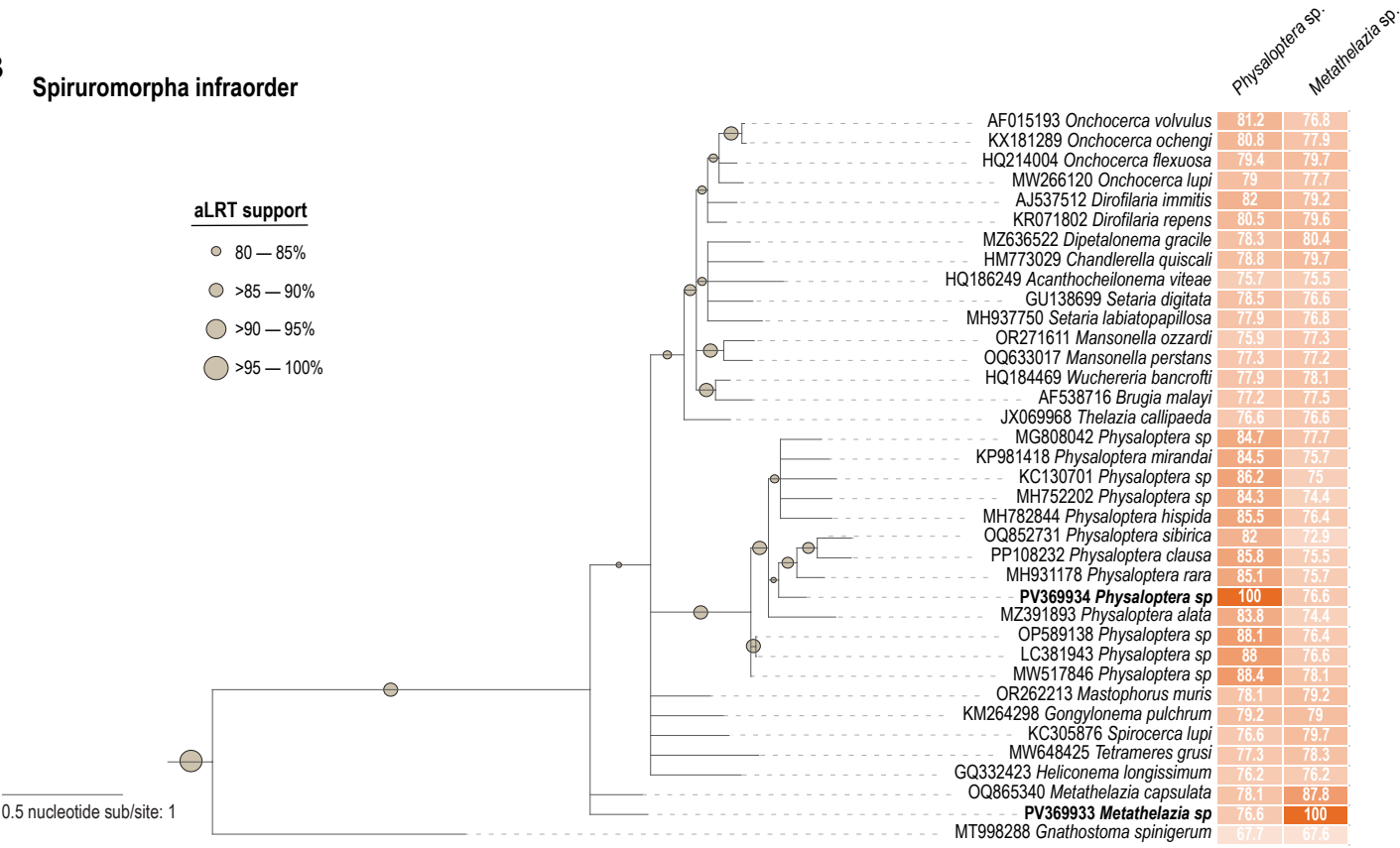

Supplement: Supplementary file 1 [file viruses-17-00745-s001.zip › viruses-3611737-supplementary/SuppFig1.pdf]
